# Supplementary material for: Unlocking the Hidden Genetic Diversity of Varicosaviruses, the Neglected Plant Rhabdoviruses
Source: Pathogens. 2022 Sep 29;11(10):1127. doi: 10.3390/pathogens11101127 (PMC9608074; doi:10.3390/pathogens11101127)

Supplementary Figure S1. Stacked bar chart showing the number of previously reported varicosaviruses and in this study.

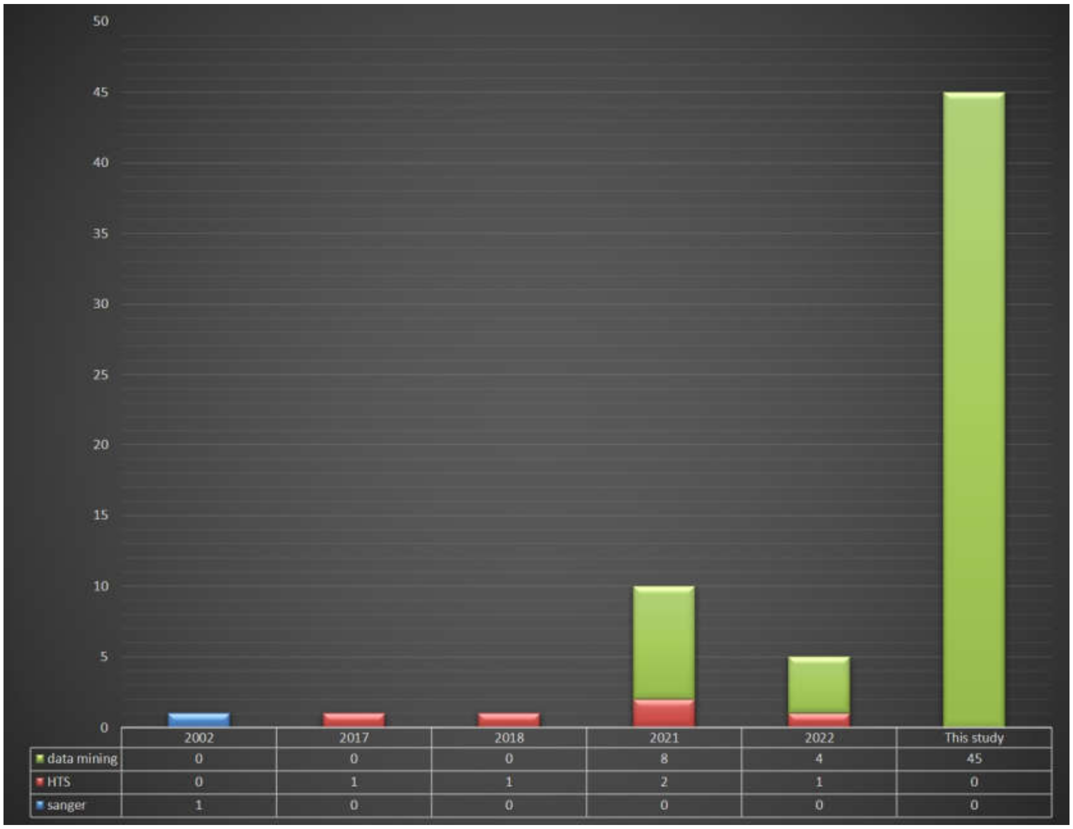

Supplement: Supplementary file 1 [file pathogens-11-01127-s001.zip › Supplementary Figure S1.pdf]
